# Association of Pesticide Exposure with Neurologic Dysfunction and Disease

**DOI:** 10.1289/ehp.7135

**Published:** 2004-05-20

**Authors:** Freya Kamel, Jane A. Hoppin

**Affiliations:** National Institute of Environmental Health Sciences, National Institutes of Health, Department of Health and Human Services, Research Triangle Park, North Carolina, USA

**Keywords:** fumigant, fungicide, insecticide, neurobehavioral performance, neurodegenerative disease, neurologic symptoms, organophosphate, Parkinson disease, pesticide

## Abstract

Poisoning by acute high-level exposure to certain pesticides has well-known neurotoxic effects, but whether chronic exposure to moderate levels of pesticides is also neurotoxic is more controversial. Most studies of moderate pesticide exposure have found increased prevalence of neurologic symptoms and changes in neurobehavioral performance, reflecting cognitive and psychomotor dysfunction. There is less evidence that moderate exposure is related to deficits in sensory or motor function or peripheral nerve conduction, but fewer studies have considered these outcomes. It is possible that the most sensitive manifestation of pesticide neurotoxicity is a general malaise lacking in specificity and related to mild cognitive dysfunction, similar to that described for Gulf War syndrome. Most studies have focused on organophosphate insecticides, but some found neuro-toxic effects from other pesticides, including fungicides, fumigants, and organochlorine and carbamate insecticides. Pesticide exposure may also be associated with increased risk of Parkinson disease; several classes of pesticides, including insecticides, herbicides, and fungicides, have been implicated. Studies of other neurodegenerative diseases are limited and inconclusive. Future studies will need to improve assessment of pesticide exposure in individuals and consider the role of genetic susceptibility. More studies of pesticides other than organophosphates are needed. Major unresolved issues include the relative importance of acute and chronic exposure, the effect of moderate exposure in the absence of poisoning, and the relationship of pesticide-related neurotoxicity to neurodegenerative disease.

Pesticides are used extensively throughout the world. In the United States, more than 18,000 products are licensed for use, and each year > 2 billion pounds of pesticides are applied to crops, homes, schools, parks, and forests [U.S. Environmental Protection Agency (EPA) Office of Pesticide Programs 2002]. Such widespread use results in pervasive human exposure.

Evidence continues to accumulate that pesticide exposure is associated with impaired health. Occupational exposure is known to result in an annual incidence of 18 cases of pesticide-related illness for every 100,000 workers in the United States (Calvert et al. 2004). The best-documented health effects involve the nervous system. The neurotoxic consequences of acute high-level pesticide exposure are well established: Exposure is associated with a range of symptoms as well as deficits in neurobehavioral performance and abnormalities in nerve function (Keifer and Mahurin 1997). Whether exposure to more moderate levels of pesticides is also neurotoxic is more controversial. Pesticide exposure may also be associated with increased risk of neurodegenerative disease, particularly Parkinson disease (Le Couteur et al. 1999).

In this review, we summarize briefly what is known about the neurotoxic effects of high-level exposure, describe in more detail the existing data on neurotoxic effects of chronic exposure at lower levels, and then discuss the relationship of pesticide exposure to neurologic disease. Although pesticide exposure may have significant effects on neurodevelopment (Eskenazi et al. 1999), this review focuses on effects in adults ≥ 18 years of age. Since differences in approach to evaluating pesticide exposure may play a crucial role in creating inconsistencies among studies, we first consider pesticide exposure assessment.

## Pesticide Exposure

Pesticides are a broad range of substances most commonly used to control insects, weeds, and fungi (plant diseases). They are frequently classified by target organism or mode of use as insecticides, herbicides, fungicides, or fumigants. Insecticides are often subclassified by chemical type as organophosphates (OPs), organochlorines, carbamates, and pyrethroids. Individuals are frequently exposed to many different pesticides or mixtures of pesticides, either simultaneously or serially. These exposures are often highly correlated, particularly within functional or chemical groups, making it difficult to identify effects of particular agents.

Studies of pesticide neurotoxicity have typically evaluated either the long-term sequelae of pesticide poisoning or the effects of occupational exposure (Table 1). Pesticide poisoning may go undiagnosed, especially among farm-workers with poor access to medical care (Moses et al. 1993) and particularly among women (London et al. 2002). Thus, workers who have never been diagnosed with pesticide poisoning may still have sustained high exposures or experienced pesticide-related illness; therefore using diagnosed poisoning as a criterion for inclusion in an exposed group or exclusion from a comparison group may incorrectly classify individuals.

Some studies of occupational pesticide exposure have classified as exposed all members of an occupational group—typically farmers or farmworkers—sometimes also considering job duration. The potential for misclassification with this approach is high. Farm owners who employ others to apply pesticides may have limited personal exposure to pesticides. Even among pesticide applicators, exposure can vary widely. For example, farmworkers with little access to information about safety practices or protective equipment (Gomes et al. 1999) may sustain far more exposure than well-trained and equipped commercial applicators (Maizlish et al. 1987). Further, farm-workers who do not apply pesticides as part of their job may still be exposed, and even family members with no direct occupational exposure may be exposed at home or elsewhere (Fenske 1997; Gladen et al. 1998), so neither of these may be an appropriate comparison group.

Factors such as application method, use of personal protective equipment, work practices related to hygiene, spills, and attitudes toward risk may all influence the degree of pesticide exposure and can be incorporated into exposure estimates (Alavanja et al. 2004; Buchanan et al. 2001; Dosemeci et al. 2002; Gomes et al. 1999; Hernandez-Valero et al. 2001; London and Myers 1998; Ohayo-Mitoko et al. 1999; Stewart et al. 2001). The relationship of these factors to exposure can be complex. For example, wearing gloves can increase exposure under some circumstances (Hines et al. 2001), perhaps because fabric (as opposed to chemically impervious) gloves can become impregnated with pesticide and serve as a reservoir of exposure. The same may be true of other types of protective clothing (Ohayo-Mitoko et al. 1999). In developing countries, use of closed pesticide mixing and loading systems may increase exposure when the equipment is used to speed up work and increase productivity rather than to protect workers (McConnell et al. 1992). Additional factors may be crucial for evaluating exposure in farmworkers, such as availability of washing and drinking water, interval between application of pesticides to a field and re-entry of workers, and housing conditions (Arcury and Quandt 1998; Gomes et al. 1999; Hernandez-Valero et al. 2001; Tielemans et al. 1999). Studies of neurotoxicity have used all these kinds of information to evaluate pesticide exposure (Gomes et al. 1999; Ohayo-Mitoko et al. 1999). The most sophisticated approaches were employed by London and Myers (1998), who used a crop-and job-specific job exposure matrix to evaluate exposure in a study of the neurotoxicity of chronic OP exposure among South African farmworkers, and by Buchanan et al. (2001), who developed an exposure algorithm to predict diazinon exposure for a study of chronic neurologic effects among sheep dippers in the United Kingdom.

Both historic and current exposures may be relevant to neurotoxicity and need to be characterized. Even among people who remain in the same occupation, current exposure may not reflect past exposure patterns because both available products and methods of use change over time. The need to evaluate past as well as current exposure has limited the utility of bio-markers; most modern pesticides are not persistent, so studies of chronic exposure rely primarily on questionnaire-based methods. Biomarkers are, however, useful in some situations. For example, organochlorines have a long half-life, so serum levels can be used as a marker of exposure to these pesticides. OP inhibition of erythrocyte acetylcholinesterase (AChE) can also be used as an exposure marker. The effect lasts 3–4 months, so AChE activity in whole blood or erythrocytes can be used to evaluate subchronic exposure, although interpretation can be complicated by acute exposure. Although the clinical utility of this biomarker in individuals may be limited by variability in baseline levels, in populations chronic OP exposure is associated with small but reliable decreases in erythrocyte AChE activity (Karr et al. 1992; Ohayo-Mitoko et al. 1997). OPs also inhibit plasma butylcholinesterase, but the effect lasts at most a few weeks and is therefore not useful for evaluating chronic exposure. Cholinesterase inhibition by carbamates lasts only minutes, so it is not a useful marker of chronic exposure to these pesticides.

Estimating lifetime pesticide exposure quantitatively is difficult because it is affected by many factors, including the multiple chemicals involved, uncertainty regarding the degree of exposure related to specific job tasks or other events, and contributions from multiple sources of exposure, including sources unrelated to occupation. Further, the biologically relevant exposure measure is not known: Peak or average exposure intensity might be more important than cumulative exposure. Thus, attempts to assess quantitative dose–response relationships may be problematic. The goal of exposure assessment in epidemiologic studies is not, however, to assign quantitative dose estimates but rather to rank individuals by relative exposure level. Assignment of either exposed or unexposed individuals to the wrong category can be a significant problem, as can combining individuals with low and high levels of exposure into one group. Random misclassification of exposure, unrelated to health outcome, will typically weaken studies by making associations more difficult to detect, although it will not undermine the validity of any association that is observed. As discussed above, assuming that all farmers or even all pesticide applicators are equally exposed is likely to entail significant misclassification, as is assuming that all farmworkers who are not applicators are not exposed. Further, studies that identify only a single highly exposed group for study cannot evaluate the neurotoxicity of moderate exposure, which may have great significance to public health. Methods described above can correctly categorize study participants with respect to their relative exposure levels, and using such methods to increase precision of exposure assessment may help minimize inconsistencies among studies.

## Neurotoxicity of High-Level Exposure

Most types of pesticides, including OP, carbamate, and organochlorine insecticides as well as fungicides and fumigants, can be neurotoxic, but only OPs have been studied in detail (Keifer and Mahurin 1997). The response to OPs can occur within minutes. Less severe cases of OP poisoning display symptoms including headache, dizziness, nausea, vomiting, pupillary constriction, and excessive sweating, tearing, and salivation. More severe cases develop muscle weakness and twitches, bronchospasm, and changes in heart rate and can progress to convulsions and coma. The mechanism of OP neurotoxicity in most cases involves overstimulation of postsynaptic cholinergic receptors after inhibition of AChE (Keifer and Mahurin, 1997), although other macromolecular targets may also be involved (Pope 1999). An intermediate syndrome, occurring 1–4 days after exposure, is characterized by muscle weakness and can be fatal if respiratory muscles are affected. Two to five weeks after exposure, some patients develop OP-induced delayed polyneuropathy, a well-characterized syndrome involving sensory abnormalities, muscle cramps, weakness, and even paralysis, primarily in the legs. These symptoms are a consequence of axonal death following OP inhibition of a neural enzyme called neuropathy target esterase and may be irreversible (Keifer and Mahurin 1997).

Several studies have shown that OP poisoning has additional long-term sequelae. Studies of individuals with a history of pesticide poisoning—farmworkers (London et al. 1998; McConnell et al. 1994; Rosenstock et al. 1991; Wesseling et al. 2002), farmers (Stallones and Beseler 2002), rescue workers (Nishiwaki et al. 2001), or individuals identified from hospitals or pesticide registries (Miranda et al. 2002; Savage et al. 1988; Steenland et al. 1994)—have found that increased symptom prevalence, deficits in cognitive and psychomotor function, decreased vibration sensitivity, and motor dysfunction can occur long after the immediate episode is resolved. In some cases, effects were observed ≥ 10 years after poisoning (Savage et al. 1988), suggesting that the residual damage is permanent. Even less severe poisoning can have long-term consequences: Banana farm workers who had been treated for intoxication with OPs or carbamates but did not require hospitalization performed worse on tests of cognitive and psychomotor function than did nonpoisoned workers when tested > 2 years later (Wesseling et al. 2002).

## Neurotoxicity of Low-Level Exposure

Findings from studies of acute exposure to moderate levels of pesticides are inconsistent. Some studies of well-trained and -equipped pesticide applicators in the United States reported that exposure to OPs sustained during a single work shift (Maizlish et al. 1987) or assessed using a short-lived urinary bio-marker (Dick et al. 2001) was associated with little neurotoxicity. However, several studies in developing countries, where exposures may have been higher, found that acute exposure to OPs was associated with increased symptom prevalence in commercial applicators (Misra et al. 1985) and farmworkers (London et al. 1998; Ohayo-Mitoko et al. 2000). Acute and chronic exposures are often correlated, sometimes making it difficult to separate their effects. The following discussion focuses on the effects of chronic exposure to moderate levels of pesticides, although in many studies acute exposure may also have occurred. Several types of neurologic end points are considered, including symptom prevalence, neurobehavioral performance, sensory and motor dysfunction, and direct measures of nerve function. Studies are summarized in Table 2.

### Symptom Prevalence

Studies of symptom prevalence are often based on variations of an established checklist (Lundberg et al. 1997) and evaluate a broad range of symptoms, including headache, dizziness, fatigue, insomnia, nausea, chest tightness, and difficulty breathing as well as symptoms suggesting cognitive (confusion, difficulty concentrating), motor (weakness, tremor), and sensory (numbness, tingling, visual disturbance) dysfunction. Pesticide exposure is associated with increases in prevalence of many symptoms, with little evidence for specificity. Most studies have focused on OPs; most of these found an association of exposure with increased symptom prevalence. Farmworkers (Gomes et al. 1998), greenhouse workers (Bazylewicz-Walczak et al. 1999), and factory workers (Bellin and Chow 1974) exposed to OPs reported increased symptom prevalence compared to unexposed workers. In particular, farmers and farmworkers who applied OPs had higher symptom prevalence than nonapplicators (London et al. 1998; Ohayo-Mitoko et al. 2000; Smit et al. 2003), as did commercial applicators (Misra et al. 1985; Steenland et al. 2000) and sheep dippers (Pilkington et al. 2001). Pesticides other than OPs also affect symptom prevalence: one study found that exposure to dichlorodiphenyl-trichloroethane (DDT) was associated with increased symptom prevalence (van Wendel de Joode et al. 2001), as did one study of fumigants (Anger et al. 1986) although not another (Calvert et al. 1998). Additional studies have evaluated changes in mood and affect, using either self-report or validated scales. Workers exposed to OPs (Bazylewicz-Walczak et al. 1999; Steenland et al. 2000; Stokes et al. 1995) or DDT (van Wendel de Joode et al. 2001) reported higher levels of tension, anger, or depression on standard symptom questionnaires, and OP applicators showed elevated levels of anxiety on personality tests (Levin et al. 1976). Three studies found no association of OPs with symptom prevalence or affect (Ames et al. 1995; Fiedler et al. 1997; Korsak and Sato 1977).

Increased symptom prevalence was correlated with inhibition of erythrocyte AChE in four studies of OP exposure (Bellin and Chow 1974; Gomes et al. 1998; Leng and Lewalter 1999; Ohayo-Mitoko et al. 2000) and with inhibition of both erythrocyte AChE and plasma cholinesterase in two of these (Bellin and Chow 1974; Leng and Lewalter 1999). Another study found no relationship of symptom prevalence to inhibition of either erythrocyte or plasma cholinesterase (Lee et al. 2003). One study found that increased symptom prevalence was associated with self-reported pesticide exposure but not with depressed erythrocyte AChE activity (Ciesielski et al. 1994). Effects of OP exposure may not necessarily be caused by AChE inhibition (Pope 1999). Further, farmworkers have complex work histories and are likely to be exposed to pesticides other than OPs that may affect symptom prevalence without affecting AChE.

### Neurobehavioral Performance

Neurobehavioral test batteries, including the World Health Organization Neurobehavioral Core Test Battery (Anger et al. 2000), the Neurobehavioral Evaluation System (Letz et al. 1996), and portions of other batteries, have been used to evaluate pesticide effects on cognitive and psychomotor function. Tests included in these batteries assess memory, attention, visuospatial processing, and other aspects of cognitive function; commonly used tests include symbol digit, digit span, visual retention, pattern memory, trail making, and others. Most studies indicate that pesticide exposure is associated with deficits in cognitive function. Sheep dippers (Stephens et al. 1995), nursery workers (Bazylewicz-Walczak et al. 1999), and other workers (Korsak and Sato 1977) exposed to OPs, malaria-control workers who sprayed DDT (van Wendel de Joode et al. 2001), vineyard workers exposed to fungicides (Baldi et al. 2001), fumigators exposed to sulfuryl fluoride but not those exposed to methyl bromide (Anger et al. 1986; Calvert et al. 1998), and farmers (Cole et al. 1997), farm-workers (Gomes et al. 1998; Kamel et al. 2003), and pesticide applicators (Farahat et al. 2003) exposed to multiple pesticides all performed worse on tests of cognitive function. There are some inconsistencies among these studies. Although most studies found deficits on one or more tests of cognitive function, different tests were affected in different studies, and a few studies found no relationship of OP exposure to any test (Ames et al. 1995; Daniell et al. 1992; Fiedler et al. 1997; Rodnitzky et al. 1975; Steenland et al. 2000).

Deficits in psychomotor function could be caused by impairment of sensory input, motor output, or associative delays; tests used include reaction time, tapping, pursuit aiming, Santa Ana and other pegboard tests, and others. Most studies indicate that pesticide exposure is associated with deficits in psychomotor function. Farmworkers (Daniell et al. 1992; London et al. 1997), farmers (Fiedler et al. 1997) and termiticide applicators (Steenland et al. 2000) exposed to OPs, malaria-control workers who sprayed DDT (van Wendel de Joode et al. 2001), vineyard workers exposed to fungicides (Baldi et al. 2001), fumigators exposed to methyl bromide or sulfuryl fluoride (Anger et al. 1986; Calvert et al. 1998), and farmworkers with multiple exposures (Gomes et al. 1998; Kamel et al. 2003) all showed worse performance on tests of psychomotor function. Again, results for individual tests were not fully consistent within or among studies, and no change in psychomotor function was evident in two studies of OP exposure (Ames et al. 1995; Cole et al. 1997).

### Sensory and Motor Dysfunction

Neurobehavioral test batteries are often supplemented with tests of sensory or motor function. One frequently used test is vibration sensitivity, which evaluates peripheral somatosensory function. Most available evidence suggests this is not affected by moderate pesticide exposure. One study of farmers exposed to OPs found decreased sensitivity (Stokes et al. 1995), and another of farmers exposed to multiple pesticides found both decreased sensitivity and other signs of peripheral neuropathy (Cole et al. 1998). However, other studies of individuals exposed to OPs (Ames et al. 1995; London et al. 1998; Pilkington et al. 2001; Steenland et al. 2000), DDT (van Wendel de Joode et al. 2001), fumigants (Anger et al. 1986; Calvert et al. 1998), or multiple pesticides (Kamel et al. 2003) found no relationship of exposure to vibration sensitivity or other measures of somatosensory function.

Few studies have evaluated other aspects of sensory function. One study suggested that the sense of smell was not affected by OPs (Steenland et al. 2000); another study suggested a relationship with fumigants (Calvert et al. 1998). Visual contrast sensitivity was not affected by exposure to OPs (Steenland et al. 2000; van Wendel de Joode et al. 2001) or multiple pesticides (Kamel et al. 2003), but color vision was (Steenland et al. 2000). Retinal degeneration was associated with fungicide exposure in a case–control study of licensed pesticide applicators (Kamel et al. 2000). In general, these data are too limited to draw conclusions about the relationship to pesticide exposure to sensory function.

Similarly, few studies have considered motor function, and few inferences can be made about its relationship to pesticide exposure. Tremor was related to exposure to multiple pesticides in one study (Davignon et al. 1965) but not to OPs in two others (London et al. 1998; Steenland et al. 2000). Grip strength was not related to exposure to fumigants (Anger et al. 1986), DDT (van Wendel de Joode et al. 2001), or multiple pesticides (Kamel et al. 2003).

Balance is an integrated sensorimotor function. An early study found deficits in balance in apple farmers exposed to multiple pesticides (Davignon et al. 1965). In modern studies, balance is commonly evaluated by a test of postural sway; varying the conditions of the test may indicate whether impaired balance is related to deficits in visual, proprioceptive, or vestibular input. Three studies of individuals exposed to OPs (Steenland et al. 2000) or to multiple pesticides (Kamel et al. 2003; Sack et al. 1993) found that impaired postural sway was associated with exposure, but effects were small and another study found no relationship of OP exposure to postural sway (Ames et al. 1995). Effects were most evident when both visual and proprioceptive inputs were removed, suggesting that vestibular function may be affected (Kamel et al. 2003; Sack et al. 1993).

### Nerve Function

Studies that have evaluated peripheral nerve conduction have produced largely negative results. Several studies of OPs found little evidence of impaired nerve conduction (Ames et al. 1995; Engel et al. 1998; Steenland et al. 2000). One study of fumigators found deficits in nerve conduction (Calvert et al. 1998), but another did not (Anger et al. 1986). In contrast, fungicide exposure was related to impaired nerve conduction in a study of bulb farmers, which also found deficits in autonomic nerve function (Ruijten et al. 1994). One study found changes in electroencephalogram (EEG) associated with OP exposure (Korsak and Sato 1977).

Three studies have performed clinical neurologic examinations in a subset of individuals identified by field studies as having deficits related to OP exposure. Beach et al. (1996) studied sheep dippers with increased symptom prevalence (Stephens et al. 1995); Horowitz et al. (1999) studied apple farmers with decreased vibration sensitivity (Stokes et al. 1995); and Jamal et al. (2002) studied sheep dippers with peripheral neuropathy (Pilkington et al. 2001). In general, clinical examination confirmed the results of the field studies, although clinically recognizable neurologic abnormalities were minor and not present in all individuals identified by the field studies.

### Genetic Susceptibility to Pesticide Neurotoxicity

Individual response to pesticide exposure may be affected by polymorphisms in genes affecting pesticide metabolism. The best-known example is paraoxonase, an enzyme that hydrolyzes active metabolites of OPs (Costa et al. 2003). Animal studies suggest that changes in serum paraoxonase activity alter susceptibility to OP toxicity (Costa et al. 2003). In humans, paraoxonase polymorphisms affect the relationship of OP exposure to both erythrocyte AChE inhibition and symptom prevalence (Lee et al. 2003; Leng and Lewalter 1999; Mackness et al. 2003; Sozmen et al. 2002). Although Costa et al. (2003) have suggested that adequate evaluation of susceptibility requires measuring serum paraoxonase activity as well as genotype, recent population-based studies have suggested that the discrepancy between genotype and phenotype is relatively small and that nongenetic factors contribute relatively little to variation in serum activity (Ferre et al. 2003; Vincent-Viry et al. 2003).

## Neurodegenerative Disease

### Parkinson Disease

An extensive literature suggests that pesticide exposure may increase risk of Parkinson disease (Le Couteur et al. 1999). Many studies have found an association of Parkinson disease risk with living in rural areas, drinking well water, and farming as an occupation (Priyadarshi et al. 2001). More specifically, case–control studies have observed that pesticide exposure is associated with increased Parkinson disease risk, although results are not fully consistent. Studies published before 1999 were reviewed by Le Couteur et al. (1999), who noted that 12 of 20 studies found a positive association, with 1.6- to 7-fold increases in risk. Some of these studies evaluated risks associated with ever exposure to any pesticide. This broad definition of exposure permits significant misclassification, which could minimize the magnitude of any association observed.

Recent studies with more detailed exposure assessment have generally found an association of pesticide exposure with Parkinson disease, with 1.5- to 7-fold increases in risk. Case–control studies found increased risk associated with possession of a pesticide use license (Baldereschi et al. 2003), cumulative pesticide exposure based on complete occupational histories (Baldi et al. 2003a; Fall et al. 1999), or occupational or other pesticide use (Herishanu et al. 2001). A cross-sectional study found an association of parkinsonism with exposure to any pesticide, although not with specific pesticides or pesticide classes (Engel et al. 2001), and an ecologic study found that Parkinson disease mortality was higher in California counties where pesticides were used than in counties where they were not (Ritz and Yu 2000). Two cohort studies with detailed exposure information confirmed these findings: Risk was related to years of plantation work and to self-reported pesticide exposure in men enrolled in the Honolulu Heart Program cohort (Petrovitch et al. 2002), and occupational exposure to pesticides assessed with a job-exposure matrix was strongly associated with Parkinson disease risk (5.6-fold increase in risk) in an older cohort living in a vineyard-growing region of France (Baldi et al. 2003b). Three case–control studies found no association of pesticide exposure with Parkinson disease (Behari et al. 2001; Kuopio et al. 1999; Taylor et al. 1999).

Most studies of pesticide exposure and Parkinson disease risk have been unable to implicate specific pesticides. Several studies found increased risk associated with exposure to either insecticides or herbicides (Butterfield et al. 1993; Gorell et al. 1998; Semchuk et al. 1992), and one study indicated that risk was elevated by exposure to organochlorines, OPs, or carbamates (Seidler et al. 1996). Several studies have implicated the herbicide paraquat (Hertzman et al. 1990; Liou et al. 1997), which produces selective degeneration of neurons involved in Parkinson disease (McCormack et al. 2002). Case reports have described Parkinson disease in individuals exposed to OPs (Bhatt et al. 1999; Davis et al. 1978); to herbicides including glyphosate (Barbosa et al. 2001), paraquat (Sanchez-Ramon et al. 1987), and diquat (Sechi et al. 1992); and to fungicides including maneb (Meco et al. 1994) and other dithiocarbamates (Hoogenraad 1988). Higher concentrations of organochlorines, particularly dieldrin, have been found in postmortem brains of Parkinson disease patients compared to patients with other neurologic diseases (Corrigan et al. 2000; Fleming et al. 1994).

Animal models have also implicated pesticide exposure in the etiology of Parkinson disease. In rats, systemic administration of rotenone has been shown to produce highly selective neural degeneration similar to that found in Parkinson disease as well as a parkinsonian behavioral disorder (Betarbet et al. 2000). Treatment of mice with both paraquat and maneb reduced motor activity and striatal tyrosine hydroxylase activity, at doses at which neither compound was effective alone (Thiruchelvam et al. 2000).

### Other Neurodegenerative Diseases

Information on pesticide exposure and other neurologic diseases is more limited. Several studies have suggested that risk of amyotrophic lateral sclerosis (ALS) is related to farming as an occupation, although not necessarily to living in rural areas (Nelson 1995–1996). Pesticide exposure has been considered in six case–control studies; three found some evidence for an association (Deapen and Henderson 1986; McGuire et al. 1997; Savettieri et al. 1991), whereas three others found none (Chancellor et al. 1993; Granieri et al. 1988; Gunnarsson et al. 1992). Only one study presented detailed exposure information (McGuire et al. 1997): Based on an industrial hygiene assessment of a complete occupational history, pesticide exposure was associated with > 2-fold increase in ALS risk, with greater risk at higher levels of exposure. This study did not implicate specific pesticides in ALS etiology. However, a cohort study found increased risk of ALS among workers exposed to the herbicide 2,4-dichlorophenoxyacetic acid (2,4-D) compared to other company employees, although this result was based on only three deaths (Burns et al. 2001). Case reports have described ALS after exposure to OPs (Bidstrup et al. 1953) and organochlorines (Fonseca et al. 1993).

Dementia has also been related to pesticide exposure. Occupational exposure to unspecified pesticides and fertilizers was associated with risk of Alzheimer disease in a large case–control study (McDowell et al. 1994), although another smaller study of environmental exposure in the general population found no relationship to herbicides, insecticides, or pesticides (Gauthier et al. 2001). Occupational exposure to any pesticide assessed with a job–exposure matrix was associated with 2-fold increase in risk of Alzheimer disease in a cohort of older individuals living in a vineyard-growing region of France and exposed primarily to dithiocarbamate fungicides (Baldi et al. 2003b). Occupational pesticide exposure was also associated with mild cognitive dysfunction in a population-based prospective study (Bosma et al. 2000), with vascular dementia (Lindsay et al. 1997), and with risk of dementia among Parkinson disease patients (Hubble et al. 1998). Understanding the relationship of pesticide exposure to Alzheimer disease may be complicated by the fact that the basic neurochemical defect in Alzheimer disease is loss of cholinergic neurons, and that to increase cholinergic tone Alzheimer disease is sometimes treated with OP cholinesterase inhibitors (Ringman and Cummings 1999).

## Conclusion

Most studies of neurotoxicity have documented an increase in symptom prevalence and changes in neurobehavioral performance reflecting cognitive and psychomotor dysfunction, but many found little effect of pesticide exposure on sensory or motor function or direct measures of nerve function. There are several potential explanations for these findings. Except for vibrotactile sensitivity, information on sensory and motor function is limited, and further study may reveal associations with pesticide exposure. Another possibility is that the increase in symptom prevalence is due to bias: Most studies were cross-sectional in design, and individuals with greater exposure or a history of poisoning may have been more motivated to recall or report symptoms. Confounding by head injury or neurologic disease, either of which might be related to both pesticide exposure and increased symptom prevalence, could also create the appearance of an association. Consistency of findings across many studies argues against these explanations, as do the positive findings of some studies that used more quantitative exposure measures. Further, bias and confounding are less likely to account for changes in neurobehavioral performance, which is assessed using objective test batteries. Thus, moderate pesticide exposure may in fact have greater effects on symptom prevalence and neurobehavioral performance than on sensory or motor function. The lack of specificity of the symptomatic response is also interesting. It is possible that the earliest or most general response to pesticide neurotoxicity is a general malaise lacking in specificity and related to mild cognitive dysfunction, similar to that described for Gulf War syndrome (White et al. 2001).

Although the weight of the evidence suggests that pesticide use is associated with increased symptom prevalence and deficits in neurobehavioral performance, there were some inconsistencies that future studies should attempt to resolve. It may be that certain functional domains are more sensitive to pesticides than others, but the current literature is too limited to resolve this question. Some of the inconsistencies among studies are likely due to methodologic differences. A critical concern is exposure assessment. Qualitative and quantitative aspects of the exposure under consideration differed among studies, as did the ability of the studies to assess exposure. Exposure measures ranged from job title to detailed assessment of cumulative exposure based on work history. There was, however, no clear-cut relationship between the quality of exposure assessment and the results of the studies.

The choice of comparison group may also influence results. Responses to symptom questionnaires and neurobehavioral performance are influenced by age, education, and cultural background (Anger et al. 1997), so it is important for comparison groups to be demographically similar to exposed populations. However, using a comparison group from the same community or workplace as the exposed participants can create problems. Although the former may have no documented exposure, they may nevertheless not be truly unexposed, limiting the power of the study to detect effects. There may be no one best solution to this problem.

Other aspects of study design, such as size, neurologic end points considered, and data analytic strategies including control for confounding, are likely to influence results. More than half of the studies considered were small, with < 100 exposed participants, and therefore had limited power to detect associations. Poor response rates in some studies may have biased results. Symptom questionnaires, neurobehavioral test batteries, and other methods for evaluating neurologic outcomes also varied among studies. In particular, different neurobehavioral batteries employ different tests of cognitive and psychomotor function. However, results were variable even for tests used in many studies. Implementation of a given test may vary between batteries; for example, a computerized version may differ from a paper-and-pencil model, but even this consideration may not explain all differences. A study of styrene found that grouping results of neurobehavioral tests provided increased power to detect effects of exposure, compared to evaluating individual tests (Heyer et al 1996). Use of similar analytic strategies might reduce inconsistencies among studies of pesticides.

Pesticide exposure may be associated with increased risk of Parkinson disease. Inconsistencies among studies are again likely to be caused by variations in study methodology, particularly lack of detailed exposure assessment in some earlier studies. The positive results from recent studies with more comprehensive exposure assessment, together with support from animal models, reinforces the hypothesis of an association. Results for ALS and Alzheimer disease are suggestive but too sparse to support firm conclusions. Whether the subtle signs of neurotoxicity found in studies of poisoning and occupational exposure are related to the later development of neurodegenerative disease is a question not adequately addressed by the literature, although one study showed that short- and long-term responses to moderate exposure are not necessarily related (Stephens et al. 1996).

Historically, most studies have focused on OPs, first to document sequelae of acute poisoning and then to explore the effects of chronic moderate exposure. There is also evidence suggesting that other types of pesticides, including organochlorines, carbamates, fungicides, and fumigants, are neurotoxic. No study has evaluated the association of herbicides with symptom prevalence or neurobehavioral performance, but these chemicals have been implicated as risk factors for Parkinson disease. Although it is important to identify classes of pesticides and even specific chemicals associated with neurotoxicity, it is also important to recognize that most workers are exposed to complex mixtures of pesticides, which may contribute synergistically to neurotoxicity.

Other aspects of the relationship of pesticide exposure to neurotoxicity remain to be clarified. Participants in most studies have sustained both chronic and acute exposures; because these are often correlated, the studies have not been able to disentangle their effects. It is also possible that studies of chronic moderate exposure have been influenced by inclusion of individuals with a history of pesticide poisoning in the exposed population. Several studies in which such individuals were excluded found no relationship of chronic exposure to neurobehavioral performance or nerve function (Ames et al. 1995; Engel et al. 1998; Fiedler et al. 1997), but other studies of nonpoisoned individuals have found associations (Kamel et al. 2003; Stephens et al. 1995; van Wendel de Joode et al. 2001), suggesting that moderate as well as high-level pesticide exposure is neurotoxic. An issue receiving increasing attention is genetic susceptibility to pesticide neurotoxicity. In particular, genetic variation in paraoxonase has been related to OP neurotoxicity.

In conclusion, there is mounting evidence that chronic moderate pesticide exposure is neurotoxic and increases risk of Parkinson disease. To substantiate these findings, future studies must employ more detailed assessment of exposure in individuals and consider the role of genetic susceptibility. More studies of pesticides other than OPs and greater attention to disentangling the effects of different types of pesticides are also needed. Better information is required to clarify the relative importance of acute and chronic exposure and the role of moderate exposure in the absence of poisoning. Finally, it will be important to clarify the relationship of pesticide-related neurotoxicity to neurodegenerative disease.

## Figures and Tables

**Table 1:** Studies of chronic pesticide exposure and neurotoxicity: exposure measurement.*^a^*

Reference	Exposed population	Chemical*^b^*	Exposure measure*^c^*	No.	Comparison group	No.
Ames et al. 1995	Pesticide registry	OP	Mild poisoning	45	Friends	90
Anger et al. 1986	Fumigators	Fumigants	High pesticide use	74	Fumigators, low exposure	29
Baldi et al. 2001	Vineyard workers	FNG	Apply pesticide	528	Farmworkers, not exposed	216
			Work in vineyards	173		
Bazylewicz-Walczak et al. 1999	Greenhouse workers	OP	Work with plants	26	Greenhouse workers, not exposed	25
Bellin and Chow 1974	Factory workers	OP, CAR	AChE inhibition	83	Faculty, students, staff	56
Calvert et al. 1998	Fumigators	Fumigants	High pesticide use	123	Friends, neighbors	120
Ciesielski et al. 1994	Farmworkers	Multiple	Self-report; AChE inhibition	202	Local population	42
Cole et al. 1997	Farmers, some applicators	OP, CAR, FNG	Apply pesticide	144	Local population	72
Cole et al. 1998	Farmers, some applicators	OP, CAR, FNG	Apply pesticide	144	Local population	72
Daniell et al. 1992	Farmworker applicators	OP	Apply pesticide	49	Slaughterhouse workers	40
Davignon et al. 1965	Apple farmers	Multiple	Apply pesticide	441	Local population	162
Engel et al. 1998	Farmworkers	OP	Current farmwork	67	Local population	68
Farahat et al. 2003	Farmworker applicators	OP, CAR, PYR	Apply pesticide	52	Clerks, administrators	50
Fiedler et al. 1997	Fruit tree farmers	OP	Cumulative exposure	57	Berry farmers; storeowners	42
Gomes et al. 1998	Farmworkers	Multiple	Past and current farmwork	226	Domestic workers	226
			Current farmwork	92		
Kamel et al. 2003	Farmworkers	Multiple	Years of work	288	Local population	51
Korsak and Sato 1977	Occupational exposure	OP	High cumulative exposure	16	Low cumulative exposure	16
Levin et al. 1976	Pesticide applicators	OP	Current pesticide use	24	Farmers	24
London et al. 1997	Fruit farm applicators	OP	Cumulative exposure	163	Farmworkers, not applicators	84
London et al. 1998	Fruit farm applicators	OP	Cumulative exposure	164	Farmworkers, not applicators	83
McConnell et al. 1994	Farmworkers	OP	Poisoning	36	Friends, siblings	36
Miranda et al. 2002	Hospital patients	OP	Poisoning	62	Cattle ranchers, fishermen	39
Misra et al. 1985	Commercial applicators	OP	Apply pesticide	22	Hospital workers	20
Nishiwaki et al. 2001	Rescue workers	OP	Poisoning	56	Rescue workers, not exposed	52
Ohayo-Mitoko et al. 2000	Farmworker applicators	OP, CAR	AChE inhibition	256	Farmworkers	152
Pilkington et al. 2001	Sheep dippers	OP	Cumulative exposure	612	Farmers, ceramic workers	160
Rodnitzky et al. 1975	Pesticide applicators	OP	Current pesticide use	23	Farmers	24
Rosenstock et al. 1991	Farmworkers	OP	Poisoning	36	Friends, siblings	36
Ruijten et al. 1994	Flower bulb farmers	FNG	Apply pesticide	131	Population	67
Sack et al. 1993	Commercial applicators	Multiple	Apply pesticide	37	Students, staff	35
Savage et al. 1988	Registry	OP	Poisoning	100	Multiple sources	100
Smit et al. 2003	Farmers	Multiple	Apply pesticide	216	Fishermen	44
Stallones and Beseler 2002	Farmers, spouses	Multiple	Poisoning	69	Other farm residents	692
Steenland et al. 1994	Pesticide registry	OP	Poisoning	128	Friends	90
Steenland et al. 2000	Commercial applicators	OP	Apply pesticide	191	Friends, blue-collar workers	189
Stephens et al. 1995	Sheep dippers	OP	Apply pesticide	146	Quarry workers	143
Stokes et al. 1995	Apple orchard applicators	OP	Apply pesticide	68	Population	68
van Wendel de Joode et al. 2001	Pesticide applicators	DDT	Years apply pesticide	27	Guards, drivers	27
Wesseling et al. 2002	Farmworkers	OP, CAR	Poisoning	81	Farmworkers	130

Abbreviations: CAR, carbamates; FNG, fungicides; PYR, pyrethroids.

**a**Only studies of chronic exposure in adults ≥ 18 years of age with comparison groups are included. Two studies (Baldi et al. 2001 and Gomes et al. 1998) evaluated two exposed groups. In four cases, two references report studies of different neurologic outcomes in the same population: Cole et al. (1997, 1998); Levin et al. (1976) and Rodnitzky et al. (1975); London et al. (1997, 1998); and McConnell et al. (1994) and Rosenstock et al. (1991). Studies are listed alphabetically.

**b**Identifies the chemical emphasized by the study; participants may have been exposed to others.

**c**Exposure measure that was used for evaluation of relationship of chronic exposure to neurotoxicity; if more than one measure was used for analysis, then the one providing the most specific information on individual exposure is listed.

**Table 2:** Studies of chronic pesticide exposure and neurotoxicity: neurologic outcomes.

Reference	Symptoms, affect	Cognitive function	Psychomotor function	Vibration sensitivity	Balance	Tremor	Nerve function*^a^*
Ames et al. 1995	0	0	0	0	0		0
Anger et al. 1986	1	1	1	0			0
Baldi et al. 2001		1	1				
Bazylewicz-Walczak et al. 1999	1	1					
Bellin and Chow 1974	1						
Calvert et al. 1998	0	1	1	0			1
Ciesielski et al. 1994	1						
Cole et al. 1997		1	0				
Cole et al. 1998				1			
Daniell et al. 1992		0	1				
Davignon et al. 1965					1	1	
Engel et al. 1998							0
Farahat et al. 2003		1					
Fiedler et al. 1997	0	0	1				
Gomes et al. 1998	1	1	1				
Kamel et al. 2003		1	1	0	1		
Korsak and Sato 1977	0	1					1
Levin et al. 1976	1						
London et al. 1997			1	0			
London et al. 1998	1			0		0	
McConnell et al. 1994				1			
Misra et al. 1985	1						
Nishiwaki et al. 2001		1	0	0	0		
Ohayo-Mitoko et al. 2000	1						
Pilkington et al. 2001	1			0			
Rodnitzky et al. 1975		0					
Rosenstock et al. 1991	1	1	1				
Ruijten et al. 1994							1
Sack et al. 1993					1		
Savage et al. 1988	1	1	1				0
Smit et al. 2003	1						
Stallones and Beseler 2002	1						
Steenland et al. 1994	1	1	1	1	0		0
Steenland et al. 2000	1	0	1	0	1	0	0
Stephens et al. 1995		1					
Stokes et al. 1995	1			1			
van Wendel de Joode et al. 2001	1	1	1	0			
Wesseling et al. 2002	1	1	1				

1 indicates the study found some relationship of pesticide exposure to the general category of outcome, although not necessarily for all tests; 0 indicates no relationship was observed for any test.

**a**Peripheral nerve conduction, EEG.
